# Lanthanum Chloride Sensitizes Cisplatin Resistance of Ovarian Cancer Cells *via* PI3K/Akt Pathway

**DOI:** 10.3389/fmed.2021.776876

**Published:** 2021-12-15

**Authors:** Shanyu Fang, Ping Zhang, Xinping Chen, Fujun Liu, Fen Wang

**Affiliations:** ^1^Department of Obstetrics and Gynecology, The First Affiliated Hospital of Nanchang University, Nanchang, China; ^2^Department of Otorhinolaryngology Head and Neck Surgery, Jiujiang University Clinical Medical College, Jiujiang University Hospital, Jiujiang, China

**Keywords:** lanthanum chloride, cisplatin resistance, ovarian cancer, PI3K, Akt

## Abstract

Our previous study manifested that lanthanum chloride (LaCl_3_) can enhance the anticancer ability of cisplatin (DDP) in ovarian cancer cells. Here, ovarian cancer cells SKOV3 and SKOV3/DDP were subjected to DDP and LaCl_3_. Cell viability, apoptosis, DNA repair, and PI3K/Akt pathway were detected. LaCl_3_ induced more cell death and apoptosis caused by DDP in two cell lines, accompanied by upregulation of Bax and Cleaved caspase 3 proteins, and downregulation of Bcl-2 protein. LaCl_3_ also could decrease RAD51 protein by inactivation of the PI3K/Akt pathway. These data indicated that LaCl_3_ could be a potential drug to modulate DDP resistance by inactivating of PI3K/Akt pathway and attenuating DNA repair in ovarian cancer.

## Introduction

Ovarian cancer is the most lethal gynecologic cancer. The first-line treatment for ovarian cancer is cisplatin (DDP)-based chemotherapy after cytoreductive surgery. However, the DDP resistance of ovarian cancer during the treatment is an important reason for the treatment failure. The 5-years survival rate is <40% (1, 2).

Cisplatin (DDP) often attacks DNA to cause DNA damage and lead to cell apoptosis; therefore, an enhanced DNA repair plays a key role in DDP resistance (3, 4). Survival pathways were necessary for cell survival and involved in chemoresistance. The PI3K/Akt survival pathway was a way to play an important role in cell survival and DDP resistance in ovarian cancer. Akt can activate the cyclin D1, NF-kB, mTOR, RAD51 or inhibit the caspase 9, p21, p27 to inhibit cell apoptosis (5–7). Lots of research target the DNA repair and PI3K/Akt pathway to explore new drugs to reverse the DDP resistance and improve the prognosis of ovarian cancer (8, 9). However, there are still no effective therapies due to the side effect. Therefore, it is important to explore novel drugs to conquer the resistance of DDP.

Lanthanum chloride (LaCl_3_) is a complex of rare earth elements. Recent studies have reported that LaCl_3_ can inhibit the proliferation and migration of cancer cells and can be an effective drug to kill cancer cells (10–12). Our previous study indicated that LaCl_3_ could augment the anticancer ability of DDP (13, 14). However, the mechanisms of LaCl_3_ acts on ovarian cancer remain unclear.

Research showed that LaCl_3_ can downregulate the PI3K/Akt signaling pathway to cause cytotoxicity; and LaCl_3_ can inactivate the Akt signaling pathway to induce autophagy (15, 16). Considering the important role of the PI3K/ Akt pathway in DDP resistance. We suggest that LaCl_3_ may reverse DDP resistance via PI3K/Akt pathway. Therefore, in this study, the relationship of LaCl_3_ and PI3K/Akt was explored using DDP sensitive and DDP resistance ovarian cancer cells. Preliminary data showed that LaCl_3_ could inactivate the PI3K/Akt pathway to inhibit DNA repair, eventually enhancing the antitumor ability of DDP in ovarian cancer.

## Materials and Methods

### Cells

Human ovarian cancer cell lines SKOV3 and SKOV3/DDP (identified by STR; Cell bank, Type Culture Collect., Chin. Sci., Shanghai, China) were cultured in RPIM-1640 (Gibco, Beijing, China) supplemented with 10% fetal bovine serum (Biological Industries, Israel), at 37°C and 5% CO_2_. SKOV3/DDP was a resistance subline of SKOV3 that grew in 0.75 μg/ml of DDP (Yunnan Phytopharm., Kunming, China). Cells were cultured with a DDP-free medium for 5 days before experiments to avoid interferences caused by residual DDP (17, 18).

### Cell Viability

Cells were seeded in a 96-well plate (5,000 cells per well) and exposed to DDP (0, 1, 2, 4, 8, 16, 32, and 64 μmol/L) or exposed to LaCl_3_ (0, 0.5, 1, 1.5, 2, 2.5, and 3 μmol/L) for 48 h. Cell viability was determined with a CCK-8 assay (MedChemExpress, United States). The half-maximal inhibition concentration (IC_50_) of DDP was calculated by the probit regression. The IC_50_ of DDP and maximum unharmful concentration of LaCl_3_ were used in the following experiments.

Subsequently, cells were subjected to DDP (IC_50_) combined with LaCl_3_ (1.5 μmol/L) for 24, 48, and 72 h, and cell viability was determined.

### Western Blot

Proteins were extracted after cells were exposed to DDP (IC_50_) and/or LaCl_3_ (1.5 μmol/L) for 48 h using RIPA buffer (Beyotime, Chongqing, China) supplemented with phenylmethanesulfonyl fluoride. Proteins were separated by sodium dodecyl sulfate-polyacrylamide gel electrophoresis and transferred to a polyvinylidene fluoride membrane (Merck Millipore, Billerica, MA). Primary antibodies as follow: anti-PI3K/p-PI3K (catalog numbers: bs-0128R, bs-332R) (Bioss Biotech., Beijing, China), anti-Akt/p-Akt (catalog numbers: 4691T, 4060T) (Cell Signaling Technology, USA), anti-Bcl2 (catalog numbers: 4223S) (Cell Signaling Technology), anti-Bax (catalog numbers: 5023S) (Bioss Biotech., Beijing, China), anti-Cleaved caspase 3 (catalog numbers: 9661T) (Cell Signaling Technology, USA), anti-RAD51 (catalog numbers: ab133534) (Abcam, UK), and anti-β-actin (catalog numbers: 66009-1-lg) (Proteintech, Wuhan, China). The secondary antibody was a goat anti-rabbit IgG antibody (catalog numbers: 7076S) (Cell Signal. Technol.). β-actin was the reference.

### Cell Apoptosis

Apoptosis cells were detected using an Annexin V assay (Keygen Biotech., Nanjing, China) after Cells were treated with DDP (IC_50_) and/or LaCl_3_ (1.5 μmol/L) for 48 h.

### Activated Assay

Cells were exposed to SC79 (an activator of Akt: 4 μg/mL;) (MedChemExpress) co-culture with DDP (IC_50_) and/or LaCl_3_ (1.5 μmol/L) for 48 h. Then CCK8 was used to detect the survival cells, and western blot was used to detect the RAD51 protein.

### Statistics

Data were processed with the software SPSS 26 (IBM, Armonk, United States). ANOVA and *t*-test were performed. The difference was significant if *p* < 0.05.

## Results

### LaCl_3_ Enhanced the Efficacy of DDP in Ovarian Cancer Cells

**Figure 1:**
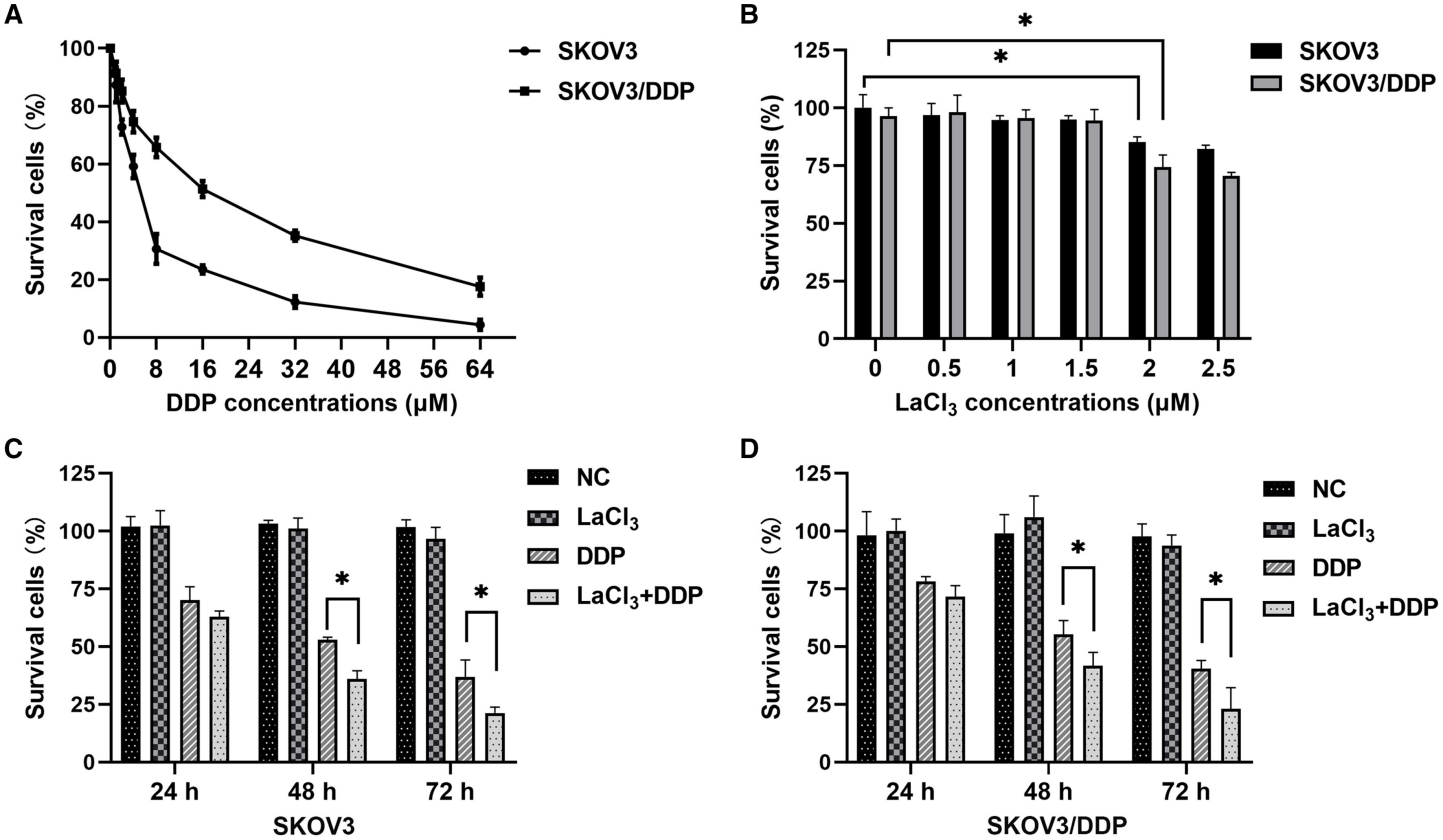
LaCl_3_ promoted cell death caused by DDP in ovarian cancer cells (*n* = 3). Percentages of survival cells after DDP treatment; higher IC_50_ values were noted and confirmed the resistance phenotype of SKOV3/DDP **(A)**. Percentages of survival cells after LaCl_3_ treatment; the percentages more than 90% after exposure to LaCl_3_ (0.5, 1, and 1.5 μmol/L), and <90% following exposure to higher concentrations of LaCl_3_ (≥2 μmol/L) **(B)**. LaCl_3_ (1.5 μmol/L) decreased the survival percentage in SKOV3 cells following DDP (5.0 μmol/L) treatment **(C)**. LaCl_3_ (1.5 μmol/L) decreased the survival percentage in SKOV3/DDP cells following DDP (15 μmol/L) treatment **(D)**. ^*^p < 0.05.

The IC_50_ values of DDP were 5 and 15 μmol/L for SKOV3 and SKOV3/DDP cells, respectively, confirming the resistance phenotype of SKOV3/DDP (Figure 1A). The percentages of survival cells were more than 90% in SKOV3 and SKOV3/DDP cells after exposure to LaCl_3_ (0.5, 1, and 1.5 μmol/L). However, the percentage of survival cells was <90% following exposure to higher concentrations of LaCl_3_ (≥ 2 μmol/L), and the percentage of dead cells in SKOV3/DDP cells was higher than in SKOV3 (*p* =0.032, *p* = 0.001) (Figure 1B). Therefore, 1.5 μmol/L of LaCl_3_ was used in the subsequent experiments.

The percentage of the dead cells was increased in SKOV3 cells following exposure to DDP combined with LaCl_3_ compared with DDP alone (*p* = 0.001–0.025) (Figure 1C). In SKOV3/DDP cells, we observed the same results (*p* = 0.013–0.026) (Figure 1D). These results indicated that LaCl_3_ enhanced the cytotoxicity of DDP.

### LaCl_3_ Enhanced Apoptosis Due to DDP

**Figure 2:**
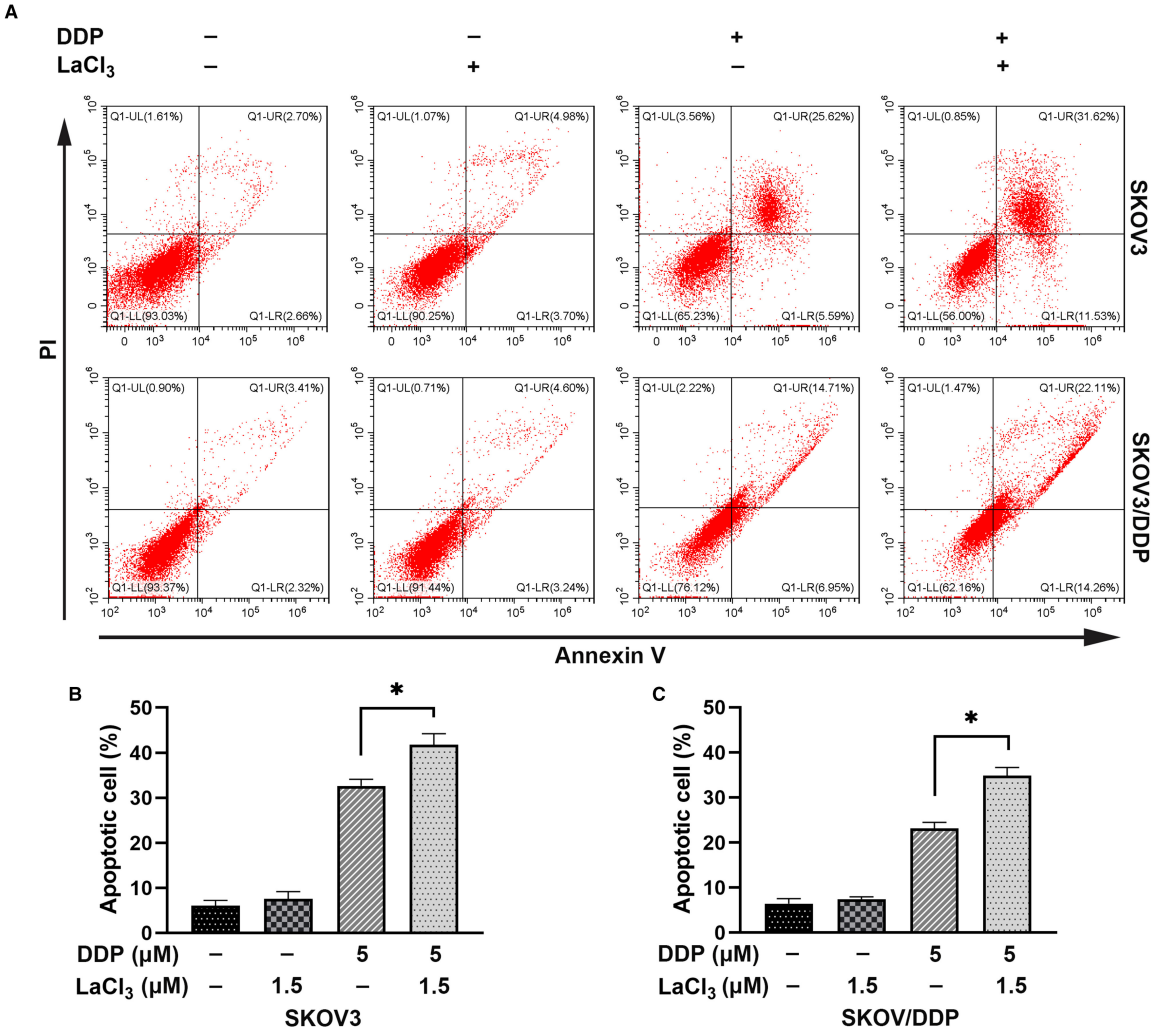
LaCl_3_ promoted cell apoptosis caused by DDP in ovarian cancer cells (*n* = 3). Apoptosis was detected by the annexin V assay; the apoptotic percentage in SKOV3 cells was increased after DDP combined with LaCl_3_ **(A,B)**. The apoptotic percentage in SKOV3/DDP cells was increased after DDP combined with LaCl_3_ **(A,C)**. *p < 0.05.

DDP caused SKOV3 and SKOV3/DDP cell apoptosis, and the combination of LaCl_3_ and DDP led to a higher percentage of apoptotic SKOV3 and SKOV3/DDP cells (*p* = 0.005, *p* = 0.001) (Figure 2).

**Figure 3:**
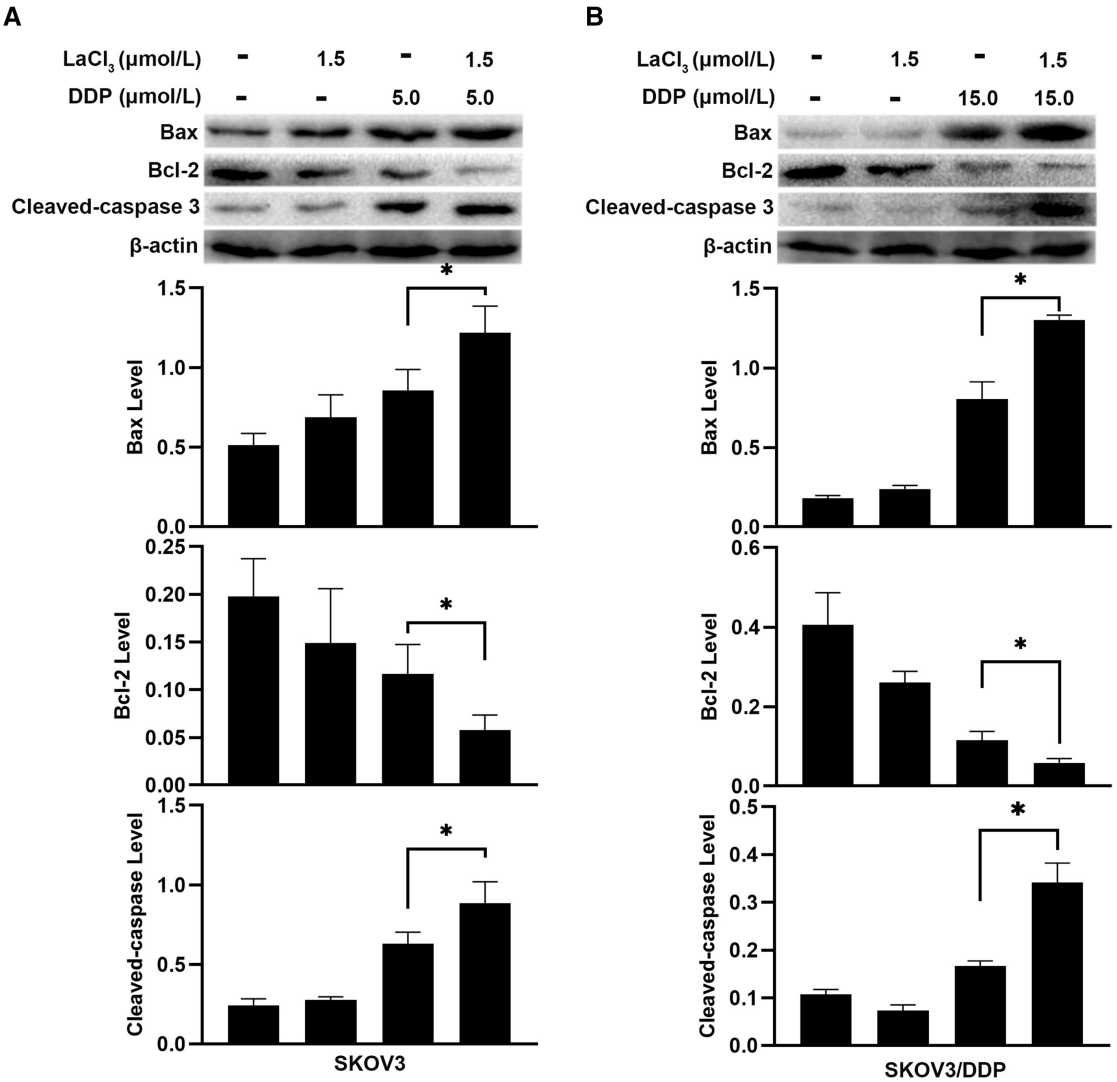
LaCl_3_ regulated apoptosis-related proteins with DDP in ovarian cancer cells (*n* = 3). DDP induced the expression of Bax and Cleaved-caspase 3, LaCl_3_ further increased them; the Bcl-2 was decreased following LaCl_3_ and DDP exposure in SKOV3 cells **(A)** and in SKOV3/DDP cells **(B)**. *p < 0.05.

Subsequently, apoptosis-related proteins Bax, Bcl-2, and Cleaved-caspase 3 were determined. In SKOV3 and SKOV3/DDP cells, the levels of Bax and Cleaved-caspase 3 were increased after treatment with LaCl_3_ and DDP (*p* = 0.045, *p* = 0.046, *p* = 0.001, and *p* = 0.002) (Figure 3); the Bcl-2 was decreased in SKOV3 and SKOV3/DDP cells after treatment with LaCl_3_ and DDP (*p* = 0.043, *p* = 0.017) (Figure 3). These data indicated that LaCl_3_ promoted cell apoptosis due to DDP both in SKOV3 and SKOV3/DDP cells.

### LaCl_3_ Inhibited DNA Repair by PI3K/Akt Pathway

**Figure 4:**
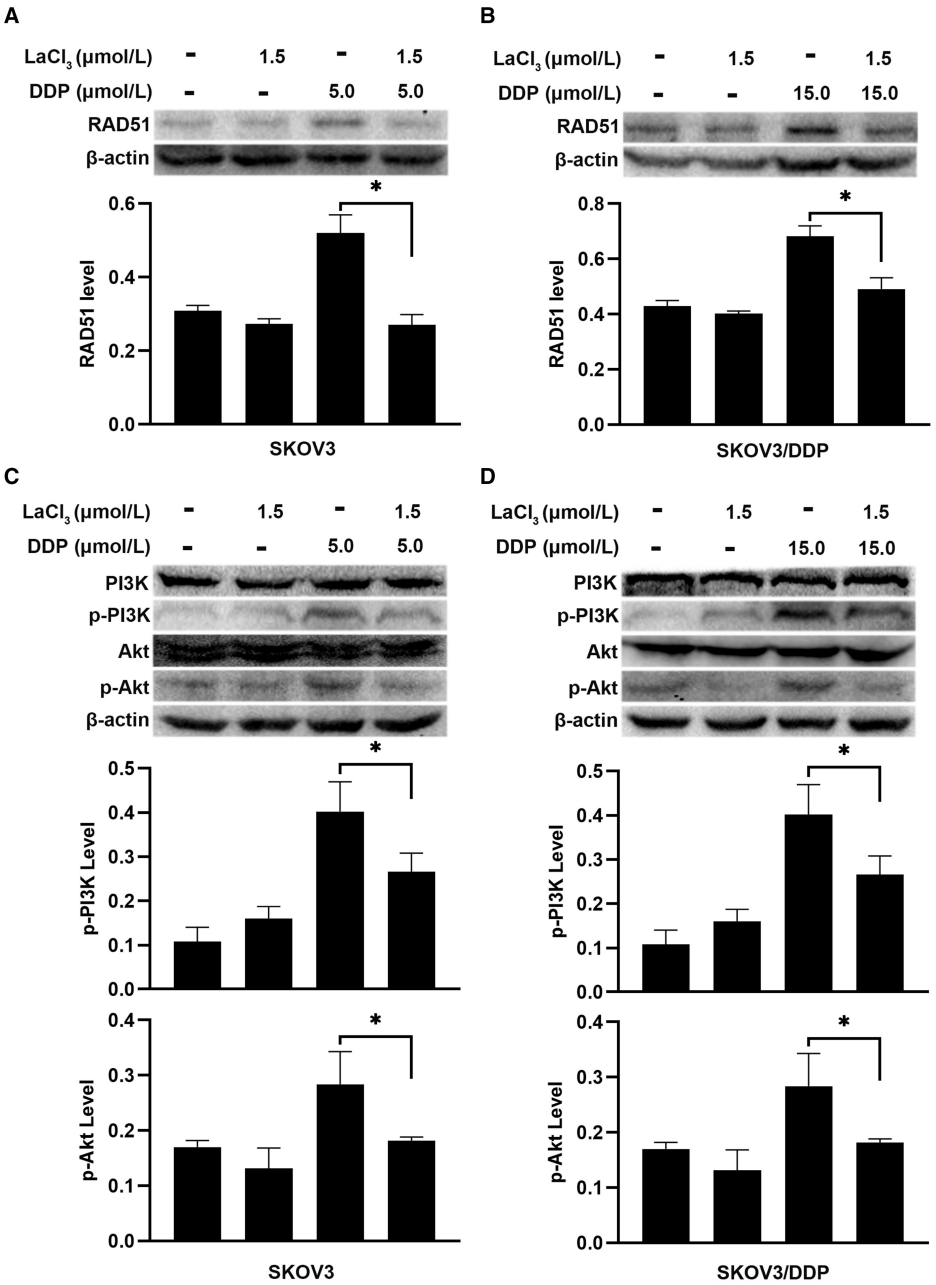
LaCl_3_ inhibited DNA repair by inactivation of PI3K/Akt pathway in ovarian cancer cells (*n* = 3). DDP induced the expression of RAD51, LaCl_3_ inhibited its expression in SKOV3 cells **(A)** and SKOV3/DDP cells **(B)**. The level of p-PI3K and p-Akt was increased after DDP exposure, and such an inductive effect was inhibited in SKOV3 cells **(C)** and SKOV3/DDP cells **(D)** following LaCl_3_ exposure. *p < 0.05.

DNA repair was assayed by detecting RAD51 since RAD51 is a key molecule for homologous recombination (HR) (19). Recent studies indicated that the PI3K/Akt can induce the expression of RAD51 (7, 20). Western blot was used to detect the expression of RAD51 and PI3K/Akt. DDP increased the RAD51 level, which means DDP not only induced DNA damage but also initiated DNA repair. However, LaCl_3_ decreased the level of RAD51 in both cell lines (*p* = 0.002, *p* = 0.004) (Figures 4A,B). DDP induced the phosphorylation of PI3K/Akt, the levels of p-PI3K and p-Akt were decreased in two cell lines following LaCl_3_ exposure (*p* = 0.04, *p* = 0.042, *p* = 0.011, *p* = 0.001) (Figures 4C,D).

**Figure 5:**
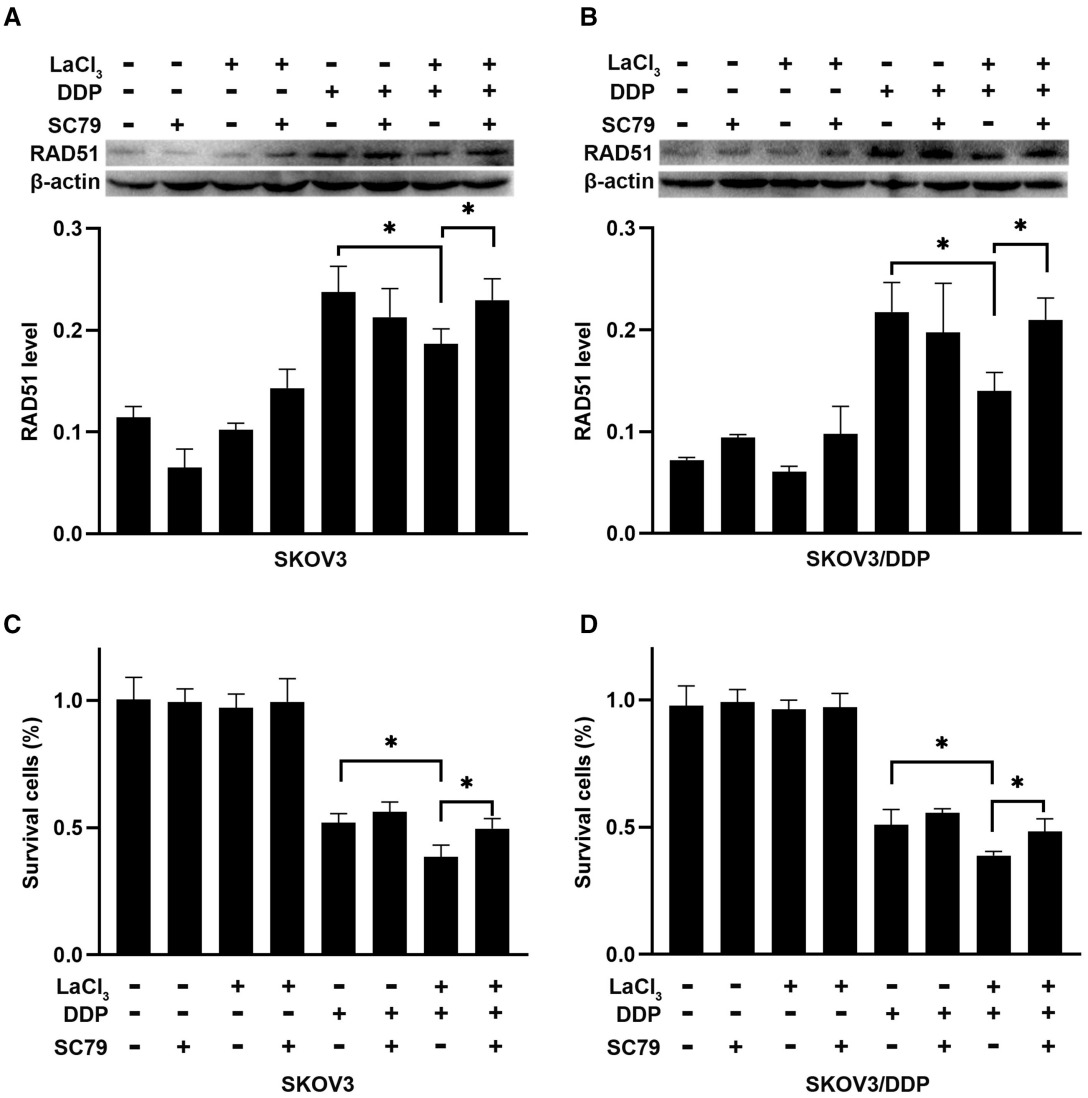
SC79 attenuated the effect of LaCl_3_ combined with DDP in ovarian cancer cells (*n* = 3). The RAD51 was upregulated in SKOV3 cells **(A)** and SKOV3/DDP cells **(B)** following SC79 (4 μg/mL) exposure. Percentages of survival cells were increased in SKOV3 cells **(C)** and SKOV3/DDP cells **(D)** following SC79 treatment. *p < 0.05.

To further demonstrate the action of PI3K/Akt on RAD51, the SC79 (an activator of Akt) was added in SKOV3 and SKOV3/DDP cells. The level of RAD51 was upregulated in both cells after exposure to SC79 (*p* = 0.045, *p* = 0.011) (Figures 5A,B). The percentages of survival cells were increased after exposure to SC79 in SKOV3 and SKOV3/DDP cells (*p* = 0.034, *p* = 0.035) (Figures 5C,D). The data indicated that LaCl_3_ could inhibit DNA repair via PI3K/Akt pathway and promote the action of DDP.

## Discussion

DDP resistance is a key obstacle for the treatment of ovarian cancer. Hence, it is of particular importance to explore new drugs to reverse DDP resistance. Here, we demonstrated that LaCl_3_ (≥2 μmol/L) caused cell death in SKOV3 and SKOV3/DDP cells. Interestingly, the percentage of dead cells in SKOV3/DDP cells was higher than in SKOV3 (*p* = 0.032, *p* = 0.001), and this needs further study to validate that the LaCl_3_ was more lethal on DDP resistant cells in the future. However, the lower concentration of LaCl_3_ (1.5 μmol/L) that was unharmful to ovarian cancer cells could increase cell death due to DDP. This was consistent with our previous study that LaCl_3_ can enhance the cytotoxicity of DDP in ovarian cancer cells (13).

Cytotoxicity of DDP was regulated by apoptosis (21, 22). Hence, enhancing cell apoptosis was the main target for drugs to reverse DDP resistance (23). The combination of DDP and LaCl_3_ led to the highest apoptotic percentage in SKOV3 and SKOV3/DDP cells. Then, the Bax and Cleaved caspase 3 were most expressed in both cells following DDP and LaCl_3_ exposure, and Bcl-2 was the least. These were consistent with the previous study that LaCl_3_ can regulate the protein expressions of Akt, Bcl-2, Bcl-xl, Bax, Bad, caspase-3, and caspase-9 to promote cell apoptosis (24); and indicated that LaCl_3_ promote cell apoptosis caused by DDP to conquer DDP resistance.

The main target of DDP is DNA, DDP attacks DNA to cause a break, and unrepair damage leads to apoptosis. RAD51 is the key protein of HR for repairing DNA damage (25, 26). In this study, the level of RAD51 was upregulated following DDP exposure, which meant DDP inducing DSB, then starting DNA repair. This possibly explains the reason that the cytotoxicity of DDP was decreased during treatment and eventually led to DDP resistance. However, the RAD51 level was decreased after LaCl_3_ exposure. These were consistent with the results of the percentages of dead and apoptotic cells following DDP and LaCl_3_ exposure and consistent with previous studies that conquer DDP resistance by inhibiting DNA repair (27). Our results primarily indicated that LaCl_3_ can enhance apoptosis by inhibiting DNA repair.

The PI3K/Akt pathway is an important survival pathway that is involved in DDP resistance in ovarian cancer (28). Here, the phosphorylation of PI3K and Akt was activated by DDP, but the inductive effect of DDP was attenuated after LaCl_3_ exposure. These results were consistent with previous studies that LaCl_3_ can inhibit the PI3K/Akt pathway to cause cytotoxicity (15, 16). Activation of Akt can increase the expression of RAD51, while inactivation of Akt downregulates the level of RAD51 to enhance cell apoptosis caused by DNA-damaging drugs (29). Hence, the SC79 (an activator of Akt) was added to neutralize the inhibiting effect of LaCl_3_, the level of RAD51 was increased that caused by DDP combined with LaCl_3_ exposure, while the percentages of dead cells were deceased. The data demonstrated that LaCl_3_ inactivated PI3K/Akt pathway to downregulate the expression of RAD51.

In conclusion, the LaCl_3_ could attenuate the DDP resistance of ovarian cancer cells via inhibiting PI3K/Akt pathway, downregulated RAD51 to inhibit DNA repair, and eventually promoted cell apoptosis due to DDP. Thus, LaCl_3_ can be a potential drug for the treatment of ovarian cancer and DDP resistance.

## Data Availability Statement

The original contributions presented in the study are included in the article/Supplementary Material, further inquiries can be directed to the corresponding author.

## Author Contributions

SF performed the experiments. SF and PZ drafted the manuscript. XC and FL checked the manuscript. FW designed the study and checked the manuscript. All authors have given approval to the final version of the manuscript.

## Funding

This work was supported by the Natural Science Foundation of Science and Technology Department of Jiangxi Province (20192BAB205076) and Natural Science Foundation of Jiangxi Province (20202BABL206101).

## Conflict of Interest

The authors declare that the research was conducted in the absence of any commercial or financial relationships that could be construed as a potential conflict of interest.

## Publisher's Note

All claims expressed in this article are solely those of the authors and do not necessarily represent those of their affiliated organizations, or those of the publisher, the editors and the reviewers. Any product that may be evaluated in this article, or claim that may be made by its manufacturer, is not guaranteed or endorsed by the publisher.
