# Supplementary material for: Lanthanum Chloride Sensitizes Cisplatin Resistance of Ovarian Cancer Cells via PI3K/Akt Pathway
Source: Front Med (Lausanne). 2021 Dec 15;8:776876. doi: 10.3389/fmed.2021.776876 (PMC8714849; doi:10.3389/fmed.2021.776876)

Figure 2

SKOV3 cells

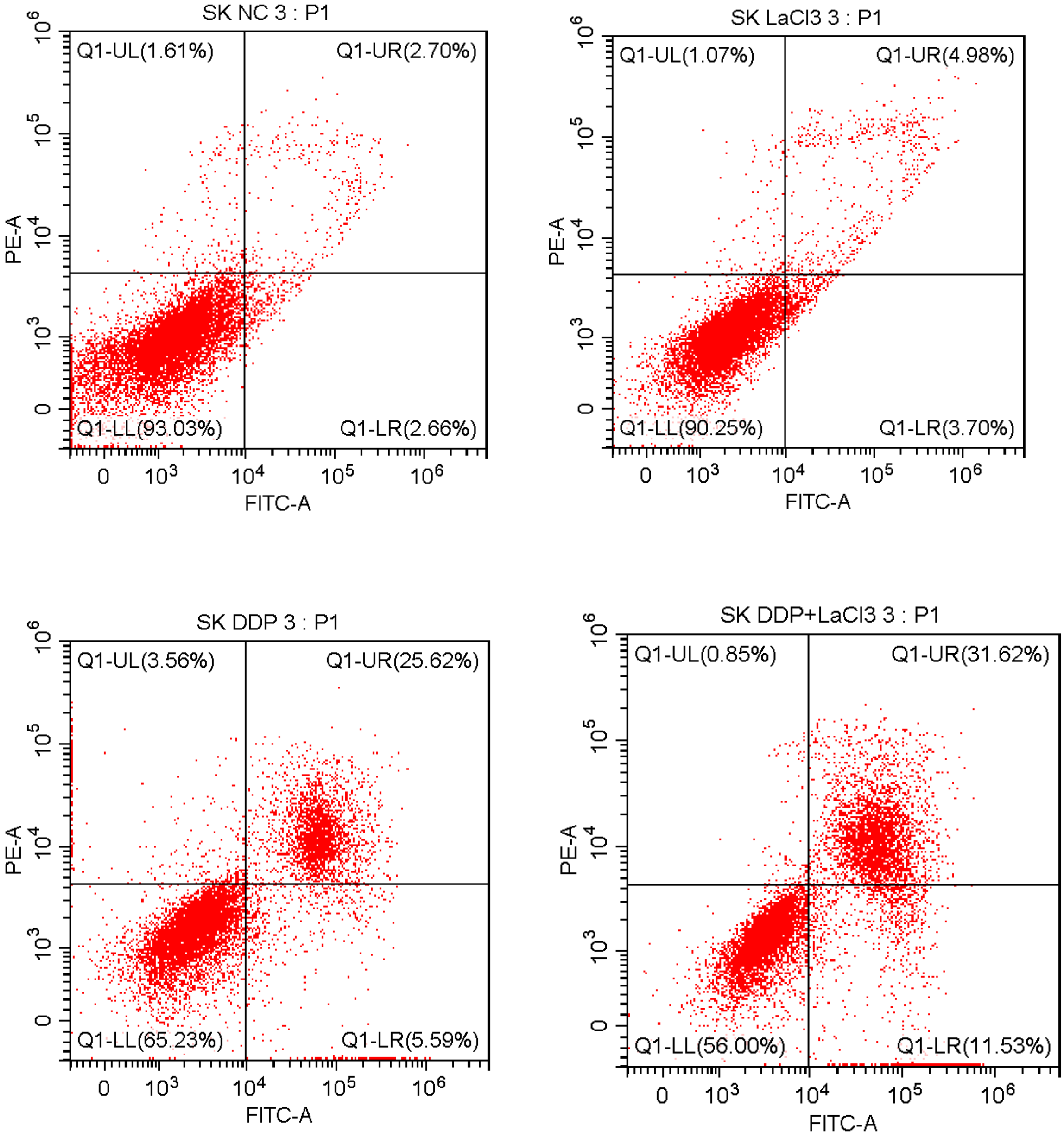

SKOV3/DDP cells

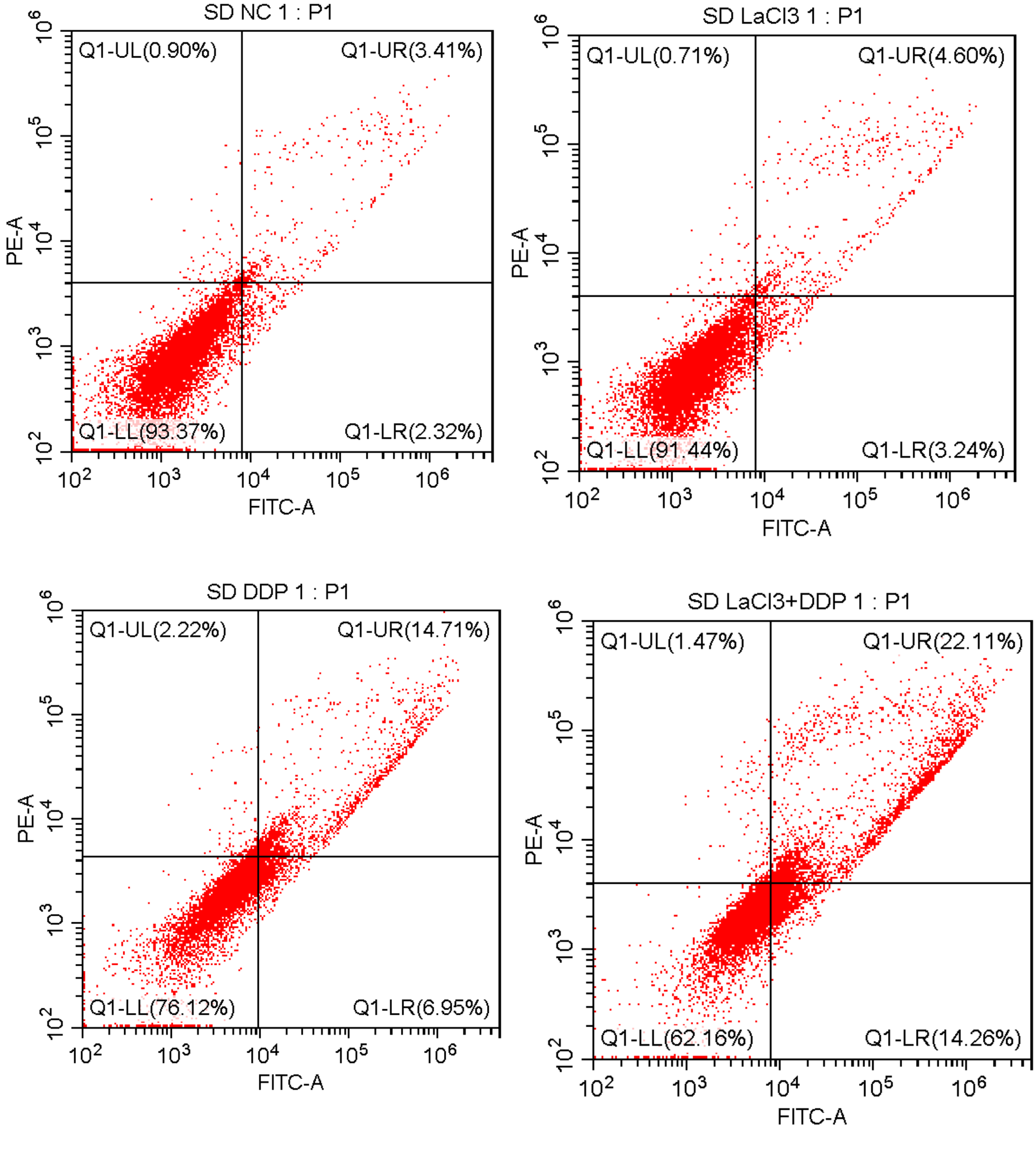

Figure 3

SKOV3 cells

SKOV3/DDP cells

Bax

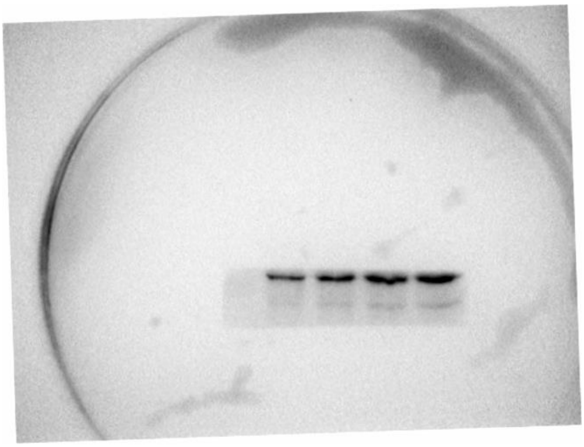

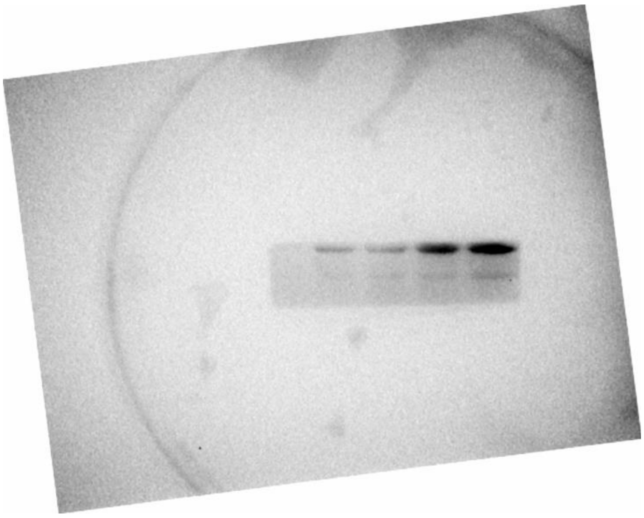

Bcl-2

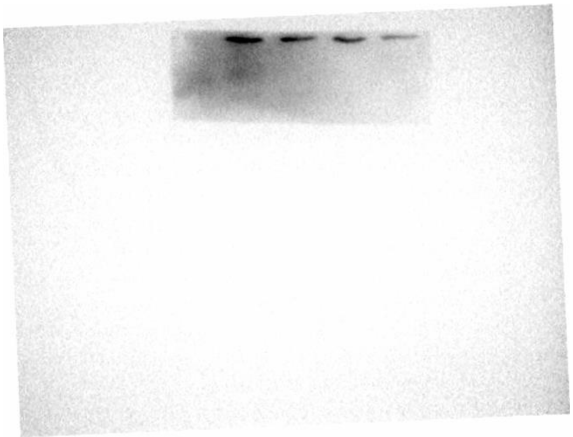

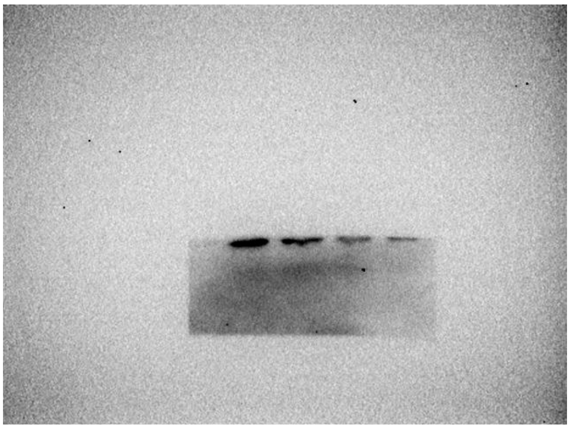

Cleaved caspase3

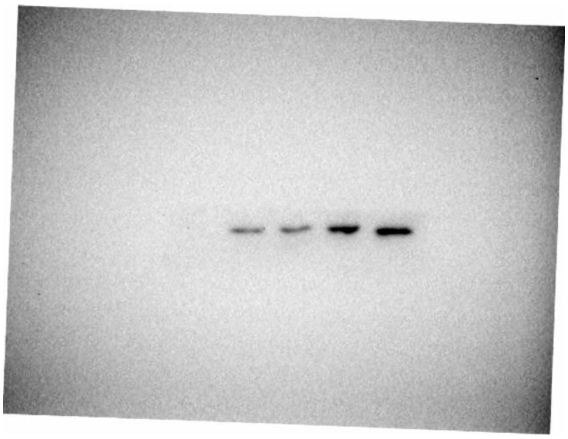

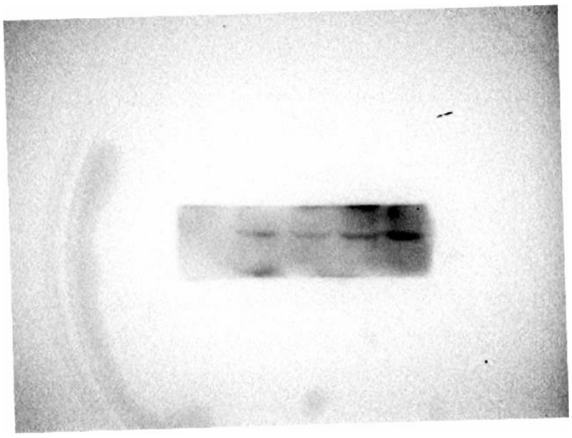

β-actin

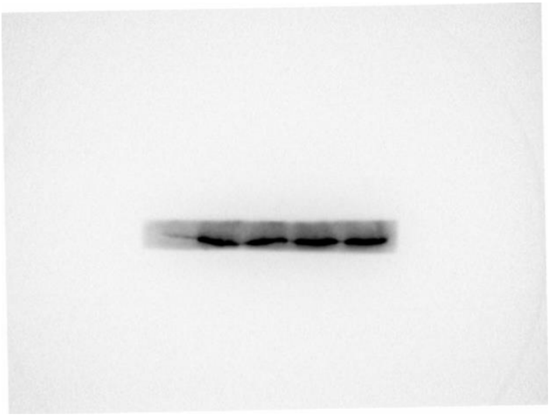

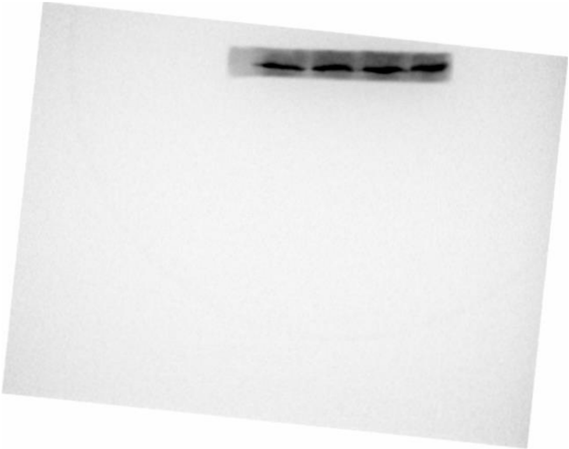

Figure 4

SKOV3 cells

SKOV3/DDP cells

RAD51

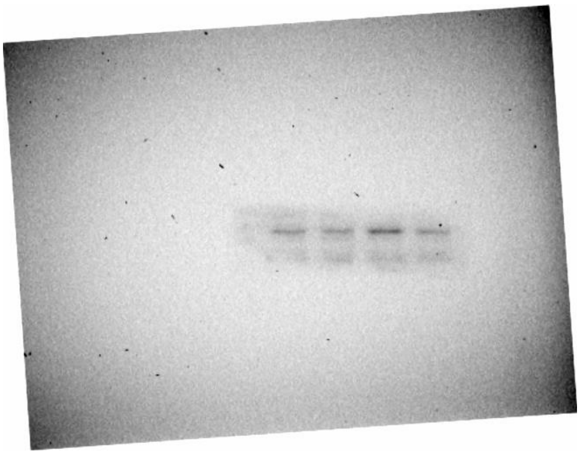

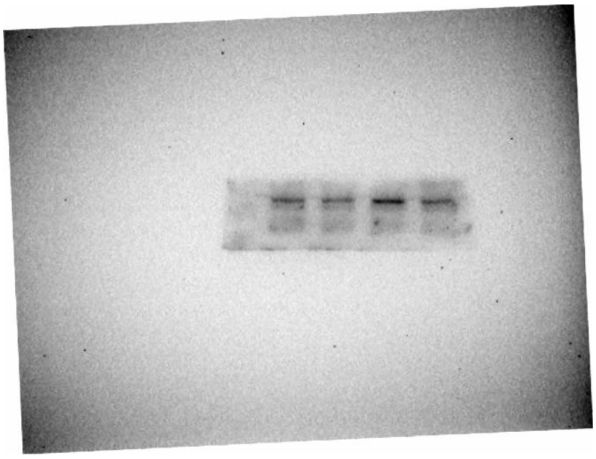

β-actin

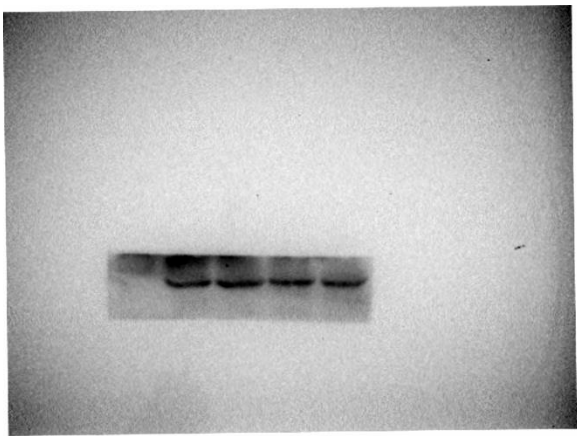

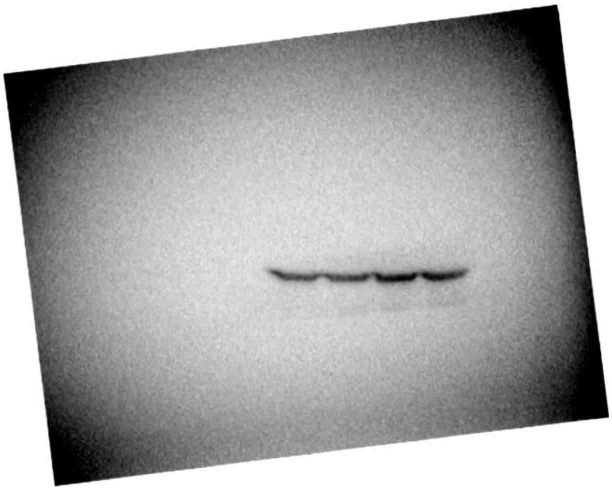

PI3K

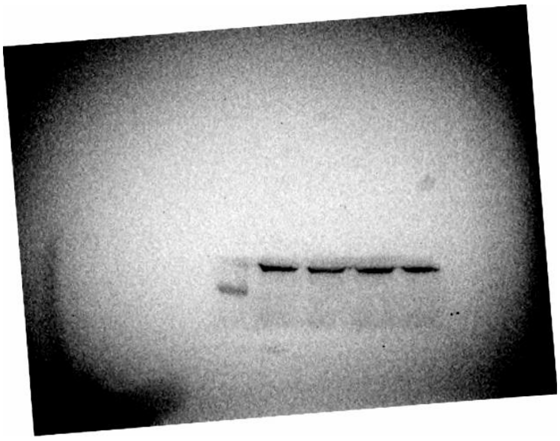

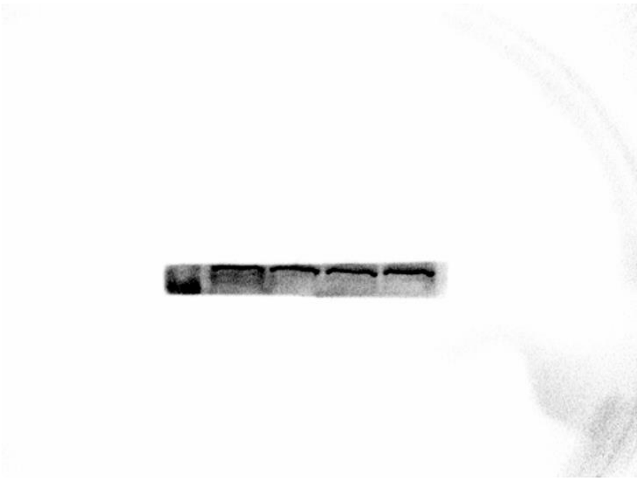

p-PI3K

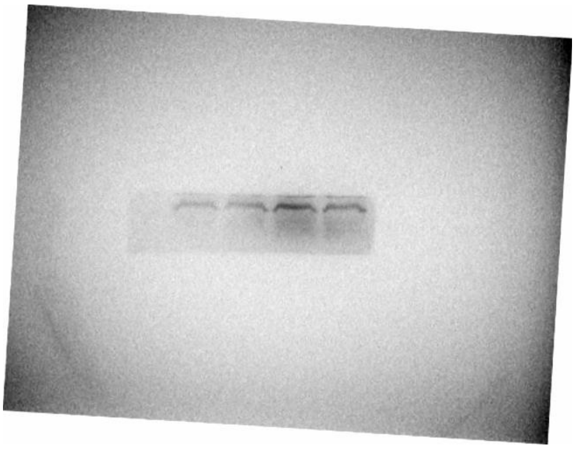

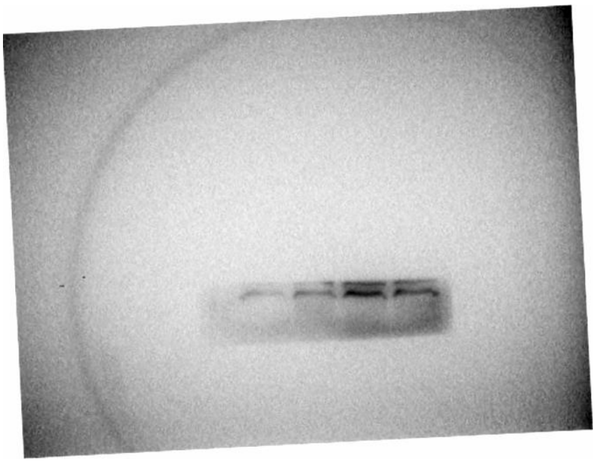

Akt

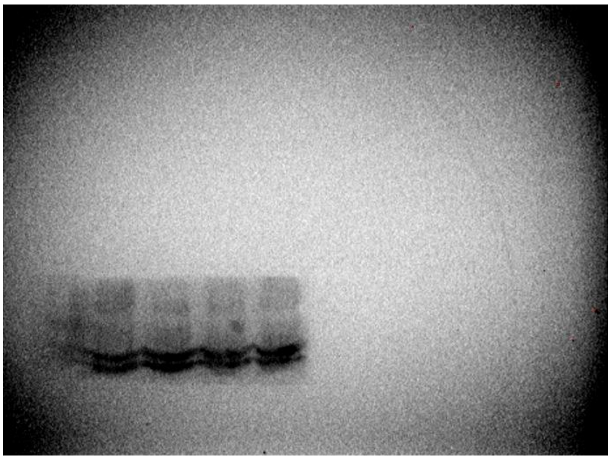

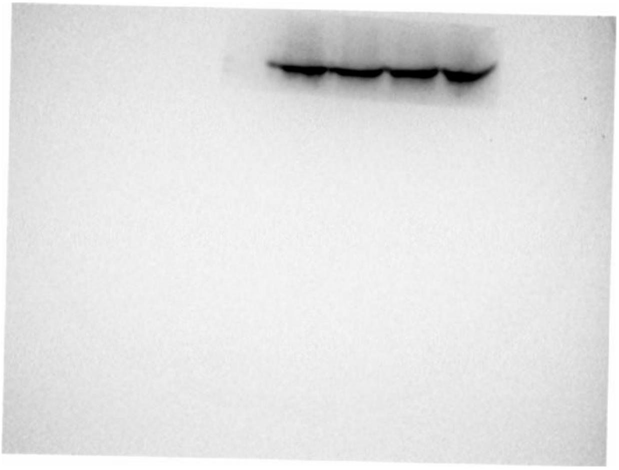

p-Akt

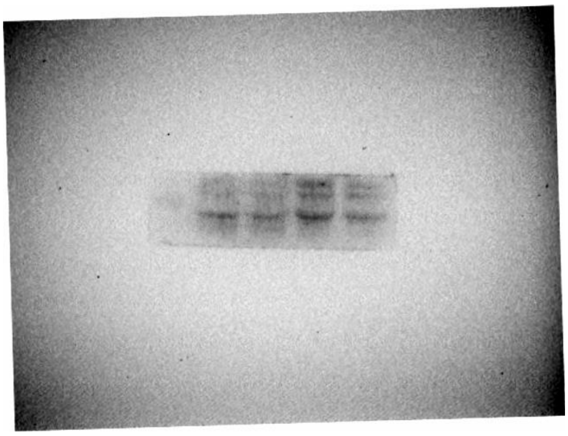 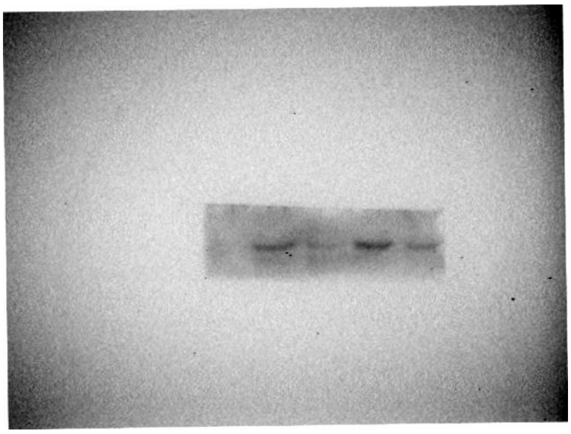

β-actin

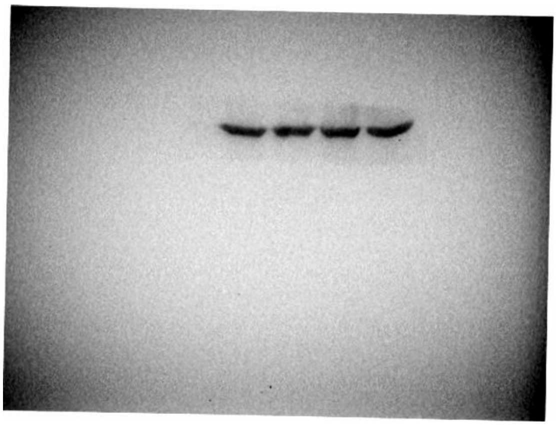 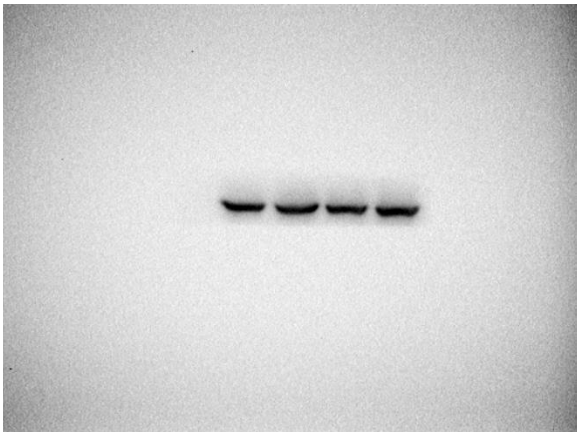

Figure 5

SKOV3 cells SKOV3/DDP cells

RAD51

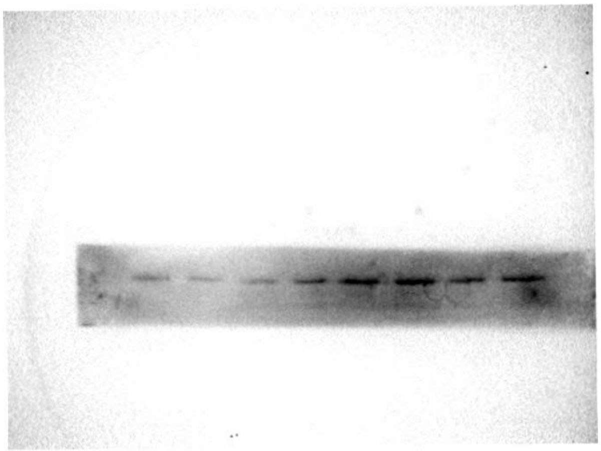 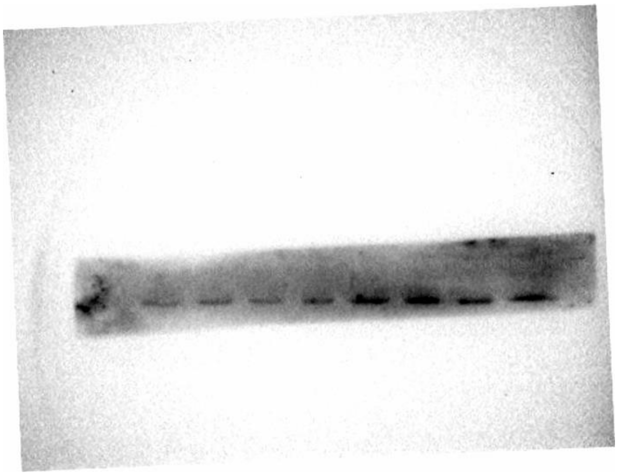

β-actin

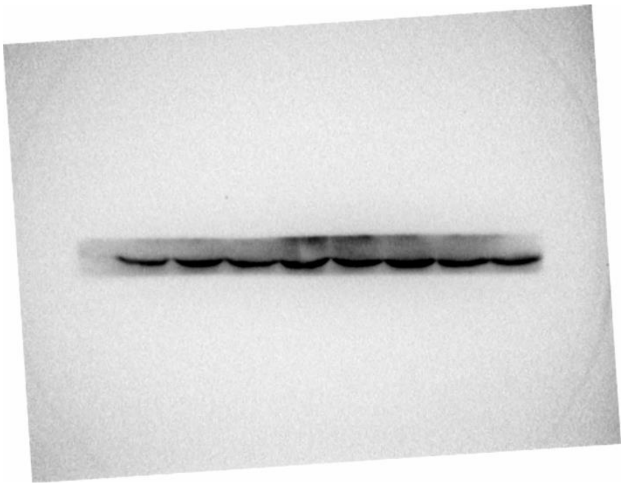

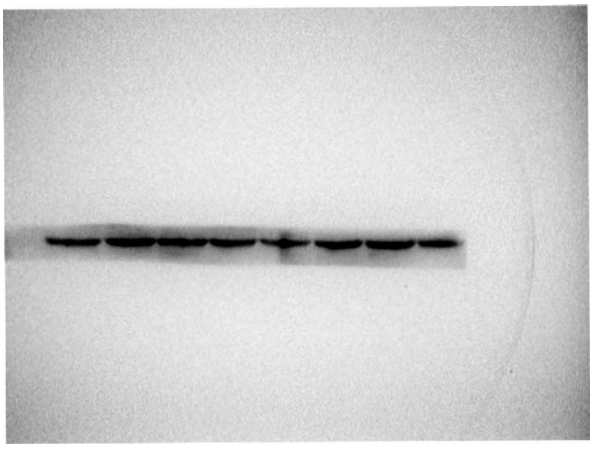

Supplement: Supplementary file 3 [file Data_Sheet_1.PDF]
